# Monkeypox as a potential emerging pandemic: a discussion on future risks and preparedness in Saudi Arabia

**DOI:** 10.3389/fpubh.2023.1254545

**Published:** 2023-11-08

**Authors:** Haneen Mohammad Shoaib

**Affiliations:** College of Business Administration, University of Business and Technology, Jeddah, Saudi Arabia

**Keywords:** Monkeypox, pandemic, health risk, health emergency, Saudi Arabia

## Abstract

Monkeypox, a zoonotic disease caused by the Monkeypox virus, has emerged as a potential threat with pandemic potential in various regions. While it is challenging to predict specific outbreaks, understanding the factors contributing to Monkeypox’s pandemic potential is important. This discussion paper explores the future risks and preparedness measures concerning Monkeypox in Saudi Arabia. The study reviews the past and current knowledge on the Monkeypox outbreak, including its clinical presentation, transmission dynamics (animals-to-humans and humans-to-human), epidemiology, and diagnostic methods. Furthermore, it explores the potential risk factors for the spread of Monkeypox within the Gulf Cooperation Council (GCC) countries, mainly in the Saudi Arabian context, considering factors such as urbanization and travel and trade patterns. The paper emphasizes the importance of early virus detection, surveillance systems, and laboratory capacity in vaccinating and responding to Monkeypox cases. Additionally, it highlights the future risks and preparedness in Saudi Arabia and the usage of social media during the pandemic seeking support and awareness about Monkeypox, and it also highlights the need for effective communication strategies of leaders through social media channels to disseminate accurate information to the public, healthcare providers, and policymakers. The discussion concludes by calling for collaborative efforts among health authorities, researchers, and international medical partners to enhance surveillance, develop outbreak response plans, and ensure the availability of vaccines and treatment options. This research serves as a foundation for guiding future preventive measures and strengthening the overall preparedness of Saudi Arabia in facing the potential emergence of Monkeypox as a future pandemic.

## 1. Monkeypox: the past and now

Monkeypox is a rare zoonotic infectious disease (1) that belongs to the genus of the Poxviridae family, which includes Orthopoxvirus, both the variola (smallpox) and the Vaccinia virus (2). Reportedly, the disease was named “Monkeypox” due to its initial discovery in monkeys in African countries (3), and after that, it has been predominantly reported in several other African countries, including Cameroon, the Central African Republic, Liberia, Nigeria, Sierra Leone, and Gabon (4). The disease occurs primarily in rural areas where people have close contact with infected animals, such as rodents or monkeys, through hunting, handling, or consumption (5). However, the Monkeypox virus can be classified into two clades. The first clade is highly contagious and unique to the Congo Basin (formerly known as Zaire) where the first case of Monkeypox was diagnosed in 1970 in a nine-month-old boy (6, 7), but at this point, studies, for example, Shaheen et al. (3) claimed that the first case of monkeypox (unknown name in that time) was reported in 1958 when outbreaks of a pox like a disease occurred in monkeys kept for research in a research laboratory in Denmark. The second clade is the West African, responsible for the last Monkeypox outbreak in the US and worldwide.

Nevertheless, Monkeypox is an infrequent infectious disease that can be transmitted from animals to humans through various means, including bites and/or scratches from infected animals (8); activities such as hunting, skinning, trapping, handling carcasses, or consuming infected animals can also lead to transmission of the virus into human (9). Unpredictably, on 10 June 2023, one case of Monkeypox from a human to a dog was reported in Paris, France (10), so still, no confirmed number of active cases of Monkeypox among animals is reported. In addition, prior studies reported that the Monkeypox virus is possibly transmitted humans-to-humans through talking or breathing closely, touching, having sex, or kissing (11), and short-range aerosols from prolonged close contact (12). Additionally, based on the Centers for Disease Control and Prevention (CDC) data, a vast majority of patients with monkeypox are among the gay or bisexual population and people working as frontline officers [e.g., healthcare workers; (13, 14)]. Also, the Monkeypox virus could be transmitted through tattoo and beauty parlor activities (15), likely from close skin-to-skin contact. Primarily, Monkeypox symptoms and signs (e.g., rash, headache, swollen lymph nodes, back pain, sore throat, high fever, and low energy level) appear within a week of transmission (16) and last for 2–4 weeks depends on the stability of the immune system of the affected individual (17). Hence, the fatality rate is usually below 10% (18).

Therefore, the first case of Monkeypox outside African countries was reported in the United States of America (United States) in 2003 (19). It is important to highlight that there have been sporadic reports of Monkeypox cases in various countries worldwide, as stated in Table 1. On 15 June 2023, the World Health Organization (WHO) reported 87,844 confirmed cases worldwide and 112 deaths from 110 countries. Thus, the situation of the Monkeypox outbreak is alarming for the next pandemic.

**Table 1:** Monkeypox cases in various countries.

Country	Confirmed cases	Deaths	Last cases updated
USA	30,404	43	24 July 2023
Brazil	10,967	16	24 July 2023
Spain	7,559	04	24 July 2023
France	4,147	00	24 July 2023
Colombia	4,090	00	24 July 2023
Mexico	4,039	30	24 July 2023
Peru	3,812	20	24 July 2023
United Kingdom	3,761	00	24 July 2023
Germany	3,691	00	24 July 2023
Total	72,470	113	

Compiled by author. Data source: Our World in Data (20).

As presented in Table 1. Monkeypox cases are instantly increasing, mainly in nine countries stepping toward a new world pandemic; bearing in mind the current situation of active cases of Monkeypox, the WHO declared Monkeypox a public health emergency of international concern on 23 July 2022 (21). To avoid the next possible global pandemic, the world’s leadership and healthcare institutions are stressed to formulate and implement several significant measures, for example, the establishment of isolation centers, tracing every infected individual, campaigns for vaccinations, educating and awareness of the people of prevention of the virus, and introduce sustain diet plans that could enhance the immune systems of the individual as to control the spread of Monkeypox at maximum level.

## 2. Monkeypox: possible next pandemic

Monkeypox has the potential to become a widely transmitted human pathogenic virus globally due to several factors. First, the scarcity of smallpox vaccination has left a significant portion of the global population without immunity to orthopoxviruses (22), increasing susceptibility to related viruses like Monkeypox. Second, Monkeypox has shown the ability to cause human-to-human transmission (11), with potential for adaptation and increased contagiousness. Furthermore, the rise in immunocompromised individuals, particularly among men who have sex with men (MSM) or bisexual, might provide a conducive environment for sustained transmission (23). Additionally, global travel and increased urbanization can facilitate its spread (24), making Monkeypox a concerning candidate for becoming a more widely transmitted human pathogen, warranting close monitoring and preparedness efforts.

However, global public health agencies like the WHO and the CDC actively monitor infectious diseases and work to prevent and control Monkeypox outbreaks globally. These agencies and local healthcare authorities are attempting to establish a sustained mechanism to respond to potential outbreaks to limit their spread. While the occurrence of a Monkeypox pandemic cannot be predicted with certainty, thus, the present research aims to explore various factors that contribute to its pandemic potential and a discussion on future risks and preparedness seeking support and awareness about Monkeypox outbreaks through social media channels (e.g., Twitter, Instagram, Facebook, and YouTube). By gaining a deeper understanding of the virus and its potential impact, this discussion contributes to preparedness efforts and informs prevention, surveillance, and control strategies.

## 3. Risk assessment of Monkeypox outbreak in the Gulf Cooperation Council countries

The Gulf Center for Disease Prevention and Control (GCDC) partnered with the WHO to address the need to evaluate the exposure risk within GCC countries’ populations and the region’s capacities in detecting and responding to the monkeypox virus. As of 2022, five out of the six countries in the GCC—Bahrain, Kuwait, Oman, Qatar, and Saudi Arabia have reported 30 Monkeypox cases (25). Despite having strong health systems, these GCC countries are vulnerable to monkeypox due to various factors, including the influx of travelers from affected regions (26), upcoming international mass gathering events (27), and the potential for ongoing transmission within the affected countries (4). Hence, the collaboration between GCDC and WHO aims to thoroughly assess the GCC countries’ population’s exposure risk and the region’s capacity to effectively detect and respond to Monkeypox outbreaks. Recently, Saudi Arabia hosted a workshop in Riyadh to address the subject of “What risk does the transmission of the Monkeypox virus pose to populations living in the GCC countries,” the key factors discussed and considered are stated below.

- Travel and Trade: The GCC countries have significant international travel and trade connections, particularly oil and gas and religious tourism, making them susceptible to the importation of infectious diseases, including monkeypox. The movement of people and goods across borders increases the chances of introducing the virus into the region, particularly if cases go undetected or appropriate preventive measures are not in place.
- Urbanization and Population Density: Urban areas in the GCC countries have high population densities, which can facilitate the rapid spread of infectious diseases. Crowded living conditions, transportation networks, and social gatherings allow the virus to circulate within communities and increase the risk of transmission.
- Migrant Worker Communities: The GCC countries have large populations of migrant workers living in communal accommodations and often in close quarters. These communities may have limited access to healthcare services and face challenges in implementing preventive measures, potentially increasing the risk of transmission within these vulnerable populations.
- Healthcare Infrastructure: While the GCC countries have well-developed healthcare systems, the transmission of monkeypox can strain healthcare facilities, particularly if there is a sudden surge in cases. Adequate resources, including healthcare personnel, diagnostic capabilities, and treatment facilities, must be available to effectively manage and contain the virus.
- Public Health Preparedness: Each GCC country’s preparedness and response capabilities level can influence the potential impact of monkeypox transmission. Strong surveillance systems, early detection mechanisms, effective contact tracing, and public health interventions are essential to control the spread of the virus and mitigate its impact on the population.

However, the GCC countries must prioritize public health measures, including surveillance, rapid diagnosis, effective infection control practices, public awareness campaigns, and vaccination strategies. Collaboration with international organizations and neighboring countries and sharing information and best practices can further enhance the region’s ability to detect, respond to, and control the transmission of Monkeypox.

### 3.1. Monkeypox outbreak in Saudi Arabia

The first case of Monkeypox was confirmed in Saudi Arabia on 14 July 2022 in Riyadh (28), and currently, 8 active cases have been reported (21, 25). However, prevention is key in controlling the spread of Monkeypox and could avoid an upcoming pandemic.

Initially, there were no specific policies and/or guidelines on controlling and monitoring the spread of the Monkeypox virus in Saudi Arabia (29). Later, the Saudi Arabian Monarchy and the Ministry of Health formulated strategies to monitor and control the spread of the virus in the country (30). Prior studies show that during the COVID-19 pandemic, the Saudi Arabian Monarchy has played a central role in coordinating and implementing measures to control the COVID-19 pandemic in the country in terms of influential leadership, policy decisions, resource allocation, public communication, vaccination campaigns, and international collaborations have contributed to efforts in reducing the spread of the virus and protecting public health (31, 32). Accordingly, the country has a response plan for emerging infectious diseases like Monkeypox (33). In a suspected or confirmed case of Monkeypox, the Ministry of Health is likely to implement preventive measures such as isolation of the infected patients, contact tracing, and monitoring of healthcare workers who may have been exposed to the virus. The Ministry also conducts public awareness campaigns to educate the public about the signs and symptoms of Monkeypox and ways to prevent its spread (34). Additionally, the Ministry of Health considered offering the smallpox vaccine to high-risk populations, such as healthcare workers and individuals traveling to areas where Monkeypox is known to occur (30).

Recently, Alshahrani et al. (35) conducted a study that aimed to know the overall knowledge level of physicians regarding the Monkeypox virus in Saudi Arabia; surprisingly, the researcher indicated that the participants showed the highest knowledge level in the areas of disease transmission 70.4% and clinical presentation 69.3%. However, they demonstrated a lower level of knowledge in prevention and control measures, 49.5%. Additionally, most physicians had a positive attitude toward preventing and controlling the Monkeypox virus, with 84.1% believing that the disease could be controlled with appropriate preventive measures. In conclusion, the study highlights the need to improve the knowledge level of physicians and other healthcare officials in Saudi Arabia regarding the Monkeypox virus, particularly in prevention and control measures. However, such practices will help in the early detection and management of cases, as well as the prevention of outbreaks.

Another empirical study by Meo et al. (29) investigated public perception, awareness, knowledge, attitudes, and acceptance of vaccination of emerging virus-like Monkeypox. The authors surveyed 1,020 participants in Riyadh, Saudi Arabia, and overall findings concluded that a significant number of individuals suggested implementing preventive measures and initiating a vaccination drive to address the Monkeypox disease. Enhancing the public’s understanding of Monkeypox and making information widely available play a crucial role in empowering the community to reduce the impact of the disease and combat viral infections both on a regional and global scale.

Considering the current outbreak of Monkeypox, Global Times reported that, in 2022, 1 million pilgrims traveled to Saudi Arabia to perform Umrah and Hajj (36), which was alarming and challenging for Saudi Arabian authorities to monitor every traveler. In this regard, during the Hajj and Umrah seasons, the Saudi Arabian government typically collaborates with international health organizations, such as the WHO, to monitor and manage any potential health risks at most of the entry points (airports, seaports, and highways) in the country (30); these measures include health screenings, vaccinations, and adherence to hygiene protocols (24). Thereby, Banjar and Alaqeel (37) pointed out that the policies and protocols for preventing the Monkeypox virus in Saudi Arabia have been formulated mainly for Hajj and Umrah Plagiarism.

**Figure 1:**
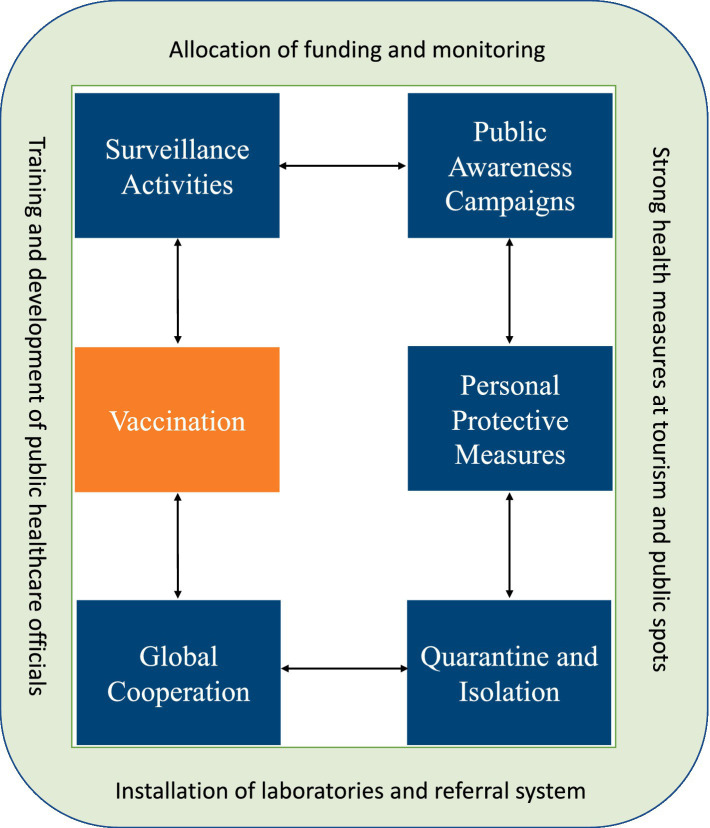
Framework for pandemic preparedness and response. Source: proposed by the author (2023).

Referring to the above discussions and arguments on the Monkeypox outbreak, the present study proposed a conceptual plan (see Figure 1) to avoid possible next pandemic preparedness and response in Saudi Arabia.

Referring to Figure 1. the strategies and preventive measures can be taken in Saudi Arabia to avoid a Monkeypox outbreak. Therefore, all the possible responses are stated below.

- Surveillance: The Saudi Arabian government should implement a surveillance system to detect any possible cases of Monkeypox early on. This system should involve monitoring travelers from areas where Monkeypox is known to occur and surveillance of suspected cases in the country.
- Quarantine and isolation: Suspected or confirmed cases of Monkeypox should be isolated to prevent the spread of the disease to others; this is particularly important in healthcare settings where the risk of transmission is higher.
- Personal protective equipment: Healthcare workers should be provided with appropriate PPE, such as gloves, gowns, and masks, to protect them from exposure to Monkeypox.
- Public awareness: The Saudi Arabian government should conduct public awareness campaigns to educate the public about the signs and symptoms of Monkeypox and ways to prevent its spread.
- Vaccination: Currently, there is no specific treatment for Monkeypox, but vaccination can effectively prevent the disease. The smallpox vaccine has been used to prevent Monkeypox with some success. The Saudi Arabian government may consider offering this vaccine to high-risk populations, such as healthcare workers and people traveling to areas where Monkeypox is known to occur.

Overall, the strategies and preventive measures for Monkeypox in Saudi Arabia should be implemented in collaboration with the WHO and other global healthcare agencies to ensure they can effectively control the spread of Monkeypox to avoid the next pandemic.

## 4. Infection prevention and control for Monkeypox

Monkeypox is a rare but potentially serious viral disease caused by the Monkeypox virus, a member of the orthopoxviral family. Prevention and control of Monkeypox require a comprehensive and coordinated approach that involves public health authorities, healthcare providers, and the general population. The following prevention and control strategies can reduce the transmission of Monkeypox.

First, the One Health Approach (OHP) for the Monkeypox virus is a comprehensive and interdisciplinary strategy that acknowledges the intricate interactions between humans, animals, and the environment in transmitting and controlling the disease (38). By recognizing the zoonotic nature of Monkeypox, this approach emphasizes the importance of collaboration and information-sharing among human health professionals, veterinarians, ecologists, and environmental experts. Furthermore, scientists can develop more accurate diagnostic tools and treatment strategies for animals and humans affected by Monkeypox through collaboration and shared resources (39).

Second, a timely vaccination is one of the most effective measures for preventing Monkeypox. The smallpox vaccine has shown cross-protection against Monkeypox and has been used in certain high-risk groups, such as healthcare workers and laboratory personnel who handle specimens (39). Studies suggest that it can provide a substantial level of immunity, reducing the severity of Monkeypox infection and lowering the risk of complications in individuals exposed to Monkeypox virus (40, 41). However, the exact level of protection may vary among individuals, and the duration of this immunity can wane over time. While it is not as effective as specific monkeypox vaccination, the smallpox vaccine remains a valuable tool in reducing the impact of Monkeypox outbreaks, particularly in regions where Monkeypox is endemic (41), and where Monkeypox-specific vaccines may be less accessible (30).

Third, early detection of Monkeypox cases is crucial for containing its spread. Healthcare providers should be educated about the clinical manifestations of the disease and be vigilant for suspected cases (34).

Fourth, strict infection control precautions in healthcare settings are essential to prevent virus transmission. This includes using personal protective equipment (PPE) like gloves, masks, gowns, and goggles and practicing proper hand hygiene. Isolation protocols should be followed for suspected or confirmed cases to minimize exposure to others.

Fifth, the most common treatments for Monkeypox primarily involve supportive care to manage symptoms and aid recovery. This includes maintaining good hygiene, wound care to prevent secondary bacterial infections (29), and the use of antiviral medications like “Cidofovir” and “Brincidofovir,” which have shown some effectiveness in reducing the severity and duration of the illness when administered early in the course of the disease (41). Additionally, pain relievers and fever reducers, such as acetaminophen or ibuprofen, may be recommended to alleviate discomfort and fever (22). In severe cases, hospitalization may be necessary to provide intravenous fluids and more intensive care. While there is no specific Monkeypox vaccine available for treatment, vaccination with the smallpox vaccine can offer some cross-protection, and it may reduce the severity of the disease in exposed individuals (41).

Sixth, identifying and monitoring individuals who had close contact with confirmed Monkeypox cases is crucial for preventing further spread. Contacts can be quarantined and monitored for the development of symptoms to prevent secondary transmission (38).

Finally, issuing travel advisories for regions experiencing Monkeypox outbreaks can help prevent international spread. Travelers can be informed about the disease and advised on necessary precautions to protect themselves and others (38).

In summary, it is crucial to recognize that the situation surrounding Monkeypox may change, and prevention and control strategies should be adapted accordingly. In this regard, public health agencies and healthcare providers must collaborate, and the public should remain informed and cooperative to prevent and control Monkeypox outbreaks effectively.

## 5. Discussion and future research studies

Conducting extensive surveillance in regions where Monkeypox is endemic and where cases have been reported outside of Africa to understand better the prevalence, geographic distribution, and potential changes in the virus (42). Conducting genomic sequencing and molecular studies to understand Monkeypox virus strains’ genetic diversity, evolution, and potential virulence factors circulating in different regions (43). This can provide insights to healthcare institutions into the virus’s ability to adapt and cause more severe disease. Therefore, developing and improving diagnostic tests, including rapid and reliable methods for the early detection and diagnosis of Monkeypox, is also important for the world (44); such practice enables healthcare institutions to avoid possible pandemics in the future. Most importantly, researching and developing effective vaccines and antiviral therapies specific to the Monkeypox outbreak (45). Enhancing our understanding of the immune response to Monkeypox infection can aid in developing vaccines and antivirals. Vaccination is crucial in preventing and controlling the spread of Monkeypox (30). The primary vaccination strategy against the Monkeypox virus is the administration of the smallpox vaccine, which provides cross-protection against monkeypox due to the similarities between the two viruses. The smallpox vaccine, known as the vaccinia vaccine, has been used historically to protect against Smallpox, which is closely related to Monkeypox. The vaccine consists of live vaccinia virus, a close relative of the Monkeypox virus, which stimulates an immune response.

However, future researchers may explore contributing to a better understanding of Monkeypox and its potential as a public health threat. However, it is important to note that the allocation of research resources depends on various factors, including the prevalence and risk assessment of the virus, as determined by health authorities and scientific organizations. If Monkeypox became the next pandemic, it could have potential implications for healthcare institutions, the education system, social activities, and economic development. Due to the novelty of the area, there is limited information available on the specific impact of a Monkeypox pandemic; some research suggestions to assess the economic burden of treating Monkeypox cases on healthcare systems, including costs related to hospitalization, diagnostics, medications, and public health interventions. This analysis could help estimate the financial resources required to manage an outbreak effectively (46). Investigating the potential impact of Monkeypox on workforce productivity due to illness, absenteeism, and reduced productivity among affected individuals or caregivers (47), in addition, this may also involve studying its short-term and long-term impacts on various sectors, including agriculture, manufacturing, services, and informal economies.

Additionally, evaluating the potential impact of a Monkeypox pandemic on international trade includes estimating the economic losses associated with travel restrictions, reduced tourism activities, and disruptions in supply chains (48). Thus, the Monkeypox pandemic could affect market confidence, investor behavior, and consumer spending patterns. Understanding an outbreak’s potential psychological and behavioral aspects can help policymakers and businesses anticipate and respond to economic fluctuations. Thus, it is important to note that these research suggestions are hypothetical, as no Monkeypox pandemic is ongoing. However, by exploring these aspects, researchers and policymakers can better understand the potential economic consequences and effectively develop strategies to mitigate and respond to any future outbreak or pandemic.

## Data availability statement

The raw data supporting the conclusions of this article will be made available by the authors, without undue reservation.

## Author contributions

HS: Conceptualization, Investigation, Writing – original draft.
